# Relationship Factors and Trajectories of Intimate Partner Violence among South African Women during Pregnancy and the Postpartum Period

**DOI:** 10.1371/journal.pone.0106829

**Published:** 2014-09-30

**Authors:** Allison K. Groves, H. Luz McNaughton-Reyes, Vangie A. Foshee, Dhayendre Moodley, Suzanne Maman

**Affiliations:** 1 Center on Health, Risk and Society, Department of Sociology, American University, Washington DC, United States of America; 2 Department of Health Behavior, University of North Carolina at Chapel Hill, Chapel Hill, North Carolina, United States of America; 3 Women's Health and HIV Research Center, Department of Obstetrics and Gynaecology University of KwaZulu-Natal, Durban, South Africa; Massachusetts General Hospital, United States of America

## Abstract

Intimate partner violence (IPV) is a significant public health problem in South Africa. However, there is limited research on whether and how IPV changes during pregnancy and the postpartum period and on the factors that might affect women's risk during this time. In this study, we describe the mean trajectories of physical and psychological IPV during pregnancy and the postpartum period and examine whether relationship power, partner social support, and relationship stress are associated with women's trajectories of IPV. Data come from a longitudinal study with 1,480 women recruited during pregnancy between May 2008 and June 2010 at a public clinic in Durban. Women completed behavioral assessments at their first antenatal visit, at fourteen weeks and at nine months postpartum. Women's experiences of IPV were measured at all three time points and relationship power, partner social support and relationship stress were each measured at the baseline assessment. We used multilevel random coefficients growth modeling to build our models. The mean trajectory for both types of IPV was flat which means that, on average, there was not significant change in levels of IPV over pregnancy and the postpartum period. However, there was significant individual variability in trajectories of IPV over the study period. Women who had higher relationship power had lower levels of physical and psychological IPV over time than women with lower relationship power. Additionally, women with higher relationship stress and lower partner support had higher levels of psychological IPV at pregnancy. Interventions that maximize women's relationship power and partner social support and minimize relationship stress during this transformative time are needed.

## Introduction

While intimate partner violence (IPV) is a major public health burden in South Africa throughout the life course [1], IPV during pregnancy and the postpartum period is of particular concern because of the negative health impacts for both women and their infants [2]–[6]. Although some researchers have studied the prevalence of IPV during pregnancy and the postpartum period, there is limited research across the globe that has examined whether and how IPV changes and what factors are associated with IPV risk during this time. Such information is crucial to the development of interventions to prevent IPV during pregnancy and the postpartum period. While some research has shown that IPV before pregnancy contributes to risk of IPV during pregnancy [7], [8]; other studies have found that women who experience IPV before or during pregnancy experience abatement of IPV in the subsequent time period [9], [10]. It is possible that the association between past and future IPV in a relationship depends on other factors in the relationship. The current study will examine change in IPV over pregnancy and the first nine months postpartum and assess whether the association between pre-pregnancy IPV and IPV during pregnancy and the postpartum period is moderated by the following three relationship factors: a woman's relationship power, or her ability to control her own decision-making, behavior and the behavior of her partner; the social support a male partner provides in the relationship; and the level of stress present within the relationship.

### Does IPV change during pregnancy and the postpartum period?

The few studies that have examined whether IPV changes as women transition to pregnancy and the postpartum period report inconsistent findings, and none of these studies take place in sub-Saharan Africa. One longitudinal study in the United States found that IPV among women increased during pregnancy and decreased in the first year postpartum [11], whereas another longitudinal study with women in four U.S. cities found that IPV did not change from pregnancy through the first six months postpartum [9]. While other cross-sectional, retrospective studies described how IPV increased for some women and decreased for others during and after pregnancy [8], [10], [12], these studies did not use statistical analyses to test whether mean levels of IPV changed across this time period, thereby limiting our ability to understand what the average pattern, or trajectory, of IPV looks like during pregnancy and postpartum.

### How history of violence within the relationship affects IPV during pregnancy and the postpartum period

In addition to understanding whether and how IPV changes during pregnancy and the postpartum period, we need more research to assess the risk and protective factors associated with IPV during this time. One such risk factor is having a history of violence within the current relationship [7], [8]. That is, women who have experienced IPV in their relationship before pregnancy are at continued risk of IPV during pregnancy and the postpartum period. On the other hand, there is also research which suggests that pregnancy and the postpartum period may be a time of respite from IPV; specifically, some women who have previously experienced IPV in their relationship find that IPV ceases or abates during pregnancy and/or postpartum [8]–[10], [12]. Taken together, these findings suggest that other factors may moderate the association between pre-pregnancy IPV and IPV during pregnancy and the postpartum period.

### How resources and experiences within the relationship may affect the association between IPV before pregnancy and IPV during pregnancy and the postpartum period

Life course theorists posit that a pattern of a behavior or outcome over time, i.e., one's trajectory, can be interrupted at developmental transitions depending on the levels of other factors such as resources and experiences that people have at that transition [13], [14]. Pregnancy is a developmental transition. Thus, levels of *resources and experiences at this developmental transition* may affect one's trajectory of IPV, or moderate the association between pre-pregnancy IPV and IPV during pregnancy and the postpartum period. Psychosocial *resources*, in particular, are posited to play “an ameliorative or positive role” at transitional moments (pg. 10 [14]). While such *resources* are broadly defined by life course theorists, violence researchers have theorized core characteristics of the relationship as resources that can particularly affect the risk of ongoing violence [15], [16]. One psychosocial *resource* that may buffer the association between past IPV and future IPV is the power a woman has to control her own decision making, her own behavior, and the behavior of her partner, so-called “relationship” power. Another psychosocial *resource* that may buffer the association between past IPV and future IPV is the social support a male partner provides within the relationship. And finally, one *experience* at the transition of pregnancy that may exacerbate the association between past IPV and future IPV may be the level of stress present within the relationship. There may be variation in these relationship factors both in relationships that are and are not violent.

### Relationship power

Women's power within the relationship may attenuate their risk of ongoing IPV. In one qualitative study, Canadian women with a history of violence in their current relationship described how their own power at a transitional moment within the relationship was a key factor that protected them from future violence [17]. Similarly, in a longitudinal cohort study, Nicaraguan women who experienced IPV at some point before or during pregnancy who had higher power within their relationship during pregnancy or postpartum were more likely to experience cessation of IPV than women who had lower relationship power [10]. These two studies both described how relationship power affected women's risk of IPV such that IPV ceased over the long term; however, they did not test whether relationship power moderated the association between past experience of IPV and future experience of IPV. Our study builds on these prior studies by testing whether relationship power weakens the association between pre-pregnancy IPV and IPV during pregnancy and the postpartum period.

### Partner social support

Another psychosocial *resource* that may buffer the association between past IPV and future IPV is the social support a male partner provides in the relationship. Criminologists Sampson and Laub posited that men's deviance (including aggression) might be interrupted at a transition like marriage. They theorized that men who were more bonded to their wives at marriage would be less likely to engage in deviance (including aggression) than men who were less bonded because they would risk losing the gains from the investments they had made in their relationship. They argued that bonding served as informal social control, and their empirical work supported their proposition [16]. As applied to this study, the continuity of IPV might be interrupted or weakened at the transition of pregnancy for women whose partners provide more social support (and are therefore more bonded and have more to lose) to their partners as compared to women whose partners provide less social support.

### Relationship stress

In addition to the role that psychosocial *resources* play in affecting trajectories at transitional moments, life course theorists posited that *experiences* at the transition might also affect trajectories [13]. However, in contrast to the potential buffering effect of psychosocial *resources*, the *experience* of relationship stress might increase the association between past IPV and future IPV. When the frequency or magnitude of relationship stress exceeds an individual's ability or perceived ability to cope in an adaptive way, negative health outcomes or behaviors (e.g., perpetration of violence) can occur [18], [19]. The transition to parenthood can be one such relationship stress for couples [20]. Couples frequently face either new or increased relationship stress at this transition and their ability to respond to this relationship stress may change interactions within the relationship. As applied to this study, the continuity of IPV might be strengthened at the transition of pregnancy in relationships where couples *experience* higher levels of relationship stress as compared to relationships where there are lower levels of relationship stress.

### The current study

There were two overarching aims in the current study: (1) to describe the mean trajectory of IPV over pregnancy and the first nine months postpartum and (2) to examine the association between pre-pregnancy IPV and trajectories of IPV during pregnancy and the postpartum period and to determine whether *resources (relationship power, partner social support)* and *experiences (relationship stress)* moderated this association. Trajectories of physical IPV and psychological IPV were modeled for both aims.

There were no a priori hypotheses for the first aim because there is no empirical research on trajectories of IPV during pregnancy and the postpartum period. There were two hypotheses for the second aim. The first hypothesis is that the positive association between pre-pregnancy IPV and IPV during pregnancy and the postpartum period would be weaker for women who reported higher relationship power and higher partner social support than for women who reported lower relationship power and lower partner support. The second hypothesis was that the positive association between pre-pregnancy IPV and IPV during pregnancy and the postpartum period would be stronger for women who reported more relationship stress than for those reporting less relationship stress. Random coefficients multilevel growth modeling was used to address study aims and test hypotheses.

## Methods

### Setting

This study was based in a public antenatal clinic in Umlazi township, which is 17 km southwest of Durban, South Africa in the province of KwaZulu Natal (KZN). Of the nine provinces in South Africa, KZN has the highest prevalence of HIV infection, the greatest proportion of individuals living in poverty and the highest infant mortality rate [21]. The Umlazi township is the second biggest township in the country [22] and is served by one public hospital (Prince Mshyieni) and seventeen public primary health clinics. [23], Women attend one of the clinics for all antenatal (ANC) visits, and they deliver their babies at Prince Mshyieni. All postnatal visits and well-baby visits are also conducted at the primary health clinic.

### Data source

The data for this analysis comes from the South Africa HIV Antenatal Post-test Support Study (SAHAPS), a longitudinal randomized controlled trial designed to provide either psychosocial support or the standard of care to 1,480 women during pregnancy and the postpartum period which took place from June 2008 through December 2011. Women who consented to participate in SAHAPS completed a baseline assessment immediately after providing written informed consent and prior to receiving clinical services (including HIV testing). After completion of the baseline survey, women were randomized to a study arm using permuted blocks of twelve, wherein subjects were randomly allocated within each block to the intervention or control condition. After randomization, women participated in the intervention or the standard of care arm from pregnancy through 10 weeks postpartum. Follow up behavioral assessments were done at 14 weeks postpartum and nine months postpartum; 1,154 women (77%) and 1,104 women (75%) returned at 4 and 9 months postpartum, respectively. All assessments were conducted by South African research assistants using computer assisted personal interviews (CAPI). The research was approved by the institutional review board at the University of North Carolina and the University of KwaZuluNatal.

### Sample

Women were recruited for SAHAPS at their first antenatal visit. Eligibility criteria included: (1) being at least 18 years old, (2) being pregnant, (3) having never tested for HIV or having tested negative for HIV at least 3 months prior to recruitment and (4) having a primary partner for at least 6 months (5) planning to bring their infant to the clinic for immunization visits, (6) being able to communicate in English or Zulu, and (7) not needing care for a high-risk pregnancy since such patients needed to be referred to a tertiary public health facility.

Of 3,333 women screened, 1,636 (49.1%) met the eligibility criteria. Of the 1,636 eligible, 1,500 (92%) women consented to participate and were subsequently enrolled and randomized. Subsequent to randomization, 13 women in the treatment arm and 7 women in the usual care arm either did not complete a clinical visit, refused HIV testing or indicated that they had tested at another location, and were therefore not eligible for the study, yielding a final baseline sample of 1,480 women.

### Measures

Measures included physical and psychological IPV, four relationship covariates (pre-pregnancy IPV, relationship power, partner social support, relationship stress) and four control covariates (age, whether the participant lived with her partner or not, assigned treatment condition and a variable called weeks-exposure). The weeks-exposure variable accounted for the number of weeks a participant had been in the study at each of the three study assessments since the time referent at each assessment was not uniform across participants. For example, some participants came in for the baseline antenatal visit when they were 20 weeks pregnant and others came in when they were 28 weeks pregnant. Similarly, the first follow up visit was scheduled for 14 weeks postpartum, though participants could complete this visit any time between 12 weeks and 8 months postpartum. Including the weeks-exposure variable in the models controlled for variation across participants in the number of weeks they had been in the study at each assessment time point. Physical IPV, psychological IPV and weeks-exposure were assessed at all three study time points. Pre-pregnancy IPV, relationship power, partner social support and relationship stress and all other control variables were taken from the baseline assessment.

A modified version of the World Health Organization (WHO) Violence Against Women instrument was used to measure IPV [24]. This instrument has ten questions on physical and psychological IPV that a woman has experienced with her current partner and has been used in numerous South African studies [25]–[27]. In this study, the stem of the questions were modified to reflect the specific reference period the participants were being asked about. For example, in the baseline survey, the questions assessed IPV before and during pregnancy. In the 14 week postpartum survey, the questions assessed IPV since delivery. In the nine month postpartum survey, the questions assessed the IPV the woman had experienced since the last time the interviewer saw them.

Physical IPV was assessed using six items from the WHO instrument. An example of one such item is, “during [reference period], how many times has your current partner pushed or shoved you?” Each item had five response categories that ranged from never to more than ten times. Each woman's response was summed across the six items to create a single measure of physical IPV (Cronbach's α = .80). The measure for physical IPV was log-transformed and a constant was added to adjust for non-normality in the distribution of the outcome.

Psychological IPV was assessed using four items from the WHO instrument. An example of one such item is, “during [reference period], how many times has your current partner insulted you or made you feel bad about yourself?” Each item had five response categories that ranged from never to more than ten times. Each woman's response was summed across the four items to create a single measure of psychological IPV (Cronbach's α = .69). The measure for psychological IPV was also log-transformed and a constant was added to adjust for non-normality in the distribution of the outcome.

Pre-pregnancy IPV was assessed using ten items from the same instrument on physical and psychological IPV. The items asked about women's experience of IPV at any time in the current relationship before pregnancy. Each woman's response was summed across the ten items and then dichotomized, where 0 = no IPV before pregnancy and 1 = one or more episodes of IPV before pregnancy.

Relationship power was assessed using twenty-two items from the modified Sexual Relationship Power scale (SRPS) [28]. One item from the original scale was dropped because it overlapped completely with the relationship stress index (“my partner might be sleeping with someone else”). The SRPS assesses power by measuring the perceived control the women has over decision-making in her relationship and by measuring the perceived control the woman has over her own behavior and her partner's behavior in the relationship. An example of one such item is “my partner does what he wants even if I do not want him to.” Each item has response categories on a Likert scale. Each woman's response was summed across the twenty-two items to create a single measure of relationship power, with higher scores being indicative of higher power for the woman within the relationship (Cronbach's α = .86).

Partner social support was assessed using seven items from the Norbeck Social Support Questionnaire, which is an instrument that assesses emotional and material support [29]. Respondents were asked to list all individuals who provided them with support in their lives and then were asked seven questions about each listed person, with response options on a five point Likert scale. A sample item measuring emotional support is “how much does this person make you feel liked or loved?” If the partner was listed, the partner social support score was the average of non-missing indicators on the scale. If the partner was not listed, the partner social support score was zero (Cronbach's α = .73).

Relationship stress was assessed by aggregating stressful events or conditions within the relationship at pregnancy (or that were directly related to the pregnancy) [30]–[32]. At baseline, the presence or absence of each of the following events or conditions were measured: unintended pregnancy, perception that one's partner was being unfaithful, the woman's report of having had multiple partners within the six months prior to pregnancy or during pregnancy, previous miscarriage, death of a child, financial stress, first-time parenthood and current legal problems An additional stressor was receiving an HIV+ diagnosis within the current pregnancy. All study participants were tested for HIV either at their first antenatal visit (following completion of the baseline survey) or at a subsequent antenatal visit. All items were summed to create a composite index of relationship stress (range 0–9).

### Data analysis

The data were first prepared for analysis by centering the variables appropriately. Following the recommendations by Raudenbush and Bryk [33], relationship power, partner social support, relationship stress and age were grand mean centered (e.g., each woman's score on relationship power was subtracted from the mean score on relationship power) to enhance interpretability of the intercept and main effects. The weeks-exposure variable was person mean centered. Finally, time denoted the point at which violence was assessed: pregnancy, 14 weeks postpartum and 9 months postpartum. It was coded as a “0” for pregnancy, “1” for 14 weeks and “2” for 9 months postpartum. Thus, the intercept can be interpreted as the level of violence at pregnancy when all other variables in the model are at the mean for the sample. All other terms were held at their means when we coded for the interaction; 0 represented the sample mean, as is the case in most interaction studies.

Second, we adjusted the sample size to exclude the 2% of participants who were missing values on any of the independent, moderating or control variables, which yielded a final sample size of 1,447 participants. Random coefficients multilevel growth curve analyses using SAS PROC MIXED [34] were used to address study aims. All analyses were specified at two levels, where time was nested within individuals.

The first step in the analysis addressed study aim 1, which was to describe the mean trajectories of physical and psychological IPV over pregnancy and the first nine months postpartum. We compared several different unconditional models that differed in functional form (flat, linear) and specifications of the random effects structures. Unconditional models examine the effect of time on the repeated measures of IPV and include no other covariates other than the weeks-exposure variable. The Bayesian Information Criterion (BIC) and multivariate Wald tests were used to determine the best-fitting model. Given the number of time points in Data Set S1(3), we tested for linear but not higher order trajectories (e.g. quadratic).

After determining the fixed and random effects that best described the trajectories of the two types of IPV, we conducted analyses to address study aim 2. The second aim was to determine if pre-pregnancy IPV and the relationship variables (relationship power, partner social support, and relationship stress) interact to influence the intercept and slope of the physical and psychological IPV trajectories after controlling for age, whether the participant lived with her partner or not, treatment condition and the weeks-exposure variable. We followed a model reduction procedure by using the Multivariate Wald test to determine if sets of interactions significantly contributed to the model (See Table 1). If a set of interactions did not significantly contribute to the model, we removed all interactions within the set regardless of whether they were significant or not. This was done to reduce Type 1 error. If the Multivariate Wald test suggested that the set did contribute to the model, then we examined the individual interactions within the set. Individual interactions that were significant were retained in the model and non-significant interactions were removed before moving to lower order terms. This procedure was followed until a final parsimonious model was obtained. The first set of interactions was the three-way interactions between pre-pregnancy IPV, the relationship variables (relationship power, partner social support, and relationship stress), and time. This set of interactions tested whether the pre-pregnancy IPV and the relationship variables interacted to influence the slopes of IPV trajectories across pregnancy and the postpartum period. The second set of interactions was the two-way interactions between each independent variable (pre-pregnancy IPV, relationship power, partner social support, and relationship stress) with time. This set of interactions tested whether each of these independent variables influenced the slopes of the IPV trajectories. The third set of interactions was the two-way interactions between pre-pregnancy IPV and each of the relationship variables (relationship power, partner social support, relationship stress). This set of interactions tested whether pre-pregnancy IPV and the relationship variables interacted to influence the intercept (IPV at pregnancy) of the trajectories.

**Table 1:** Sets of interactions tested.

Model	Component tested	Interactions of interest	Wald test (X^2^)physical	Wald test (X^2^) psych	df	Conclusion
Set 1	determine if the interactions between pre-pregnancy IPV and the three relationship variables influenced the slopes of the trajectories	pre-pregnancy IPV x relationship power x time	2.4	6.5	3	Wald test not significant, all three-way interactions dropped from model
		pre-pregnancy IPV x partner social support x time				
		pre-pregnancy IPV x relationship stress x time				
Set 2	determine if the main effects of pre-pregnancy IPV and the three relationship variables influenced the slope of the trajectories	pre-pregnancy IPV x time	14.9**	11.6*	4	Wald test significant; significant interactions retained in model
		relationship power x time				
		partner social support x time				
		relationship stress x time				
Set 3	determine if the interactions between pre-pregnancy IPV and the three relationship variables influenced the intercept of the trajectories	pre-pregnancy IPV*relationship power	35.2***	31.5***	3	Wald test significant; significant interactions retained in model
		pre-pregnancy IPV*partner social support				
		pre-pregnancy IPV*relationship stress				

*p<.05; **p<.01; ***p<.001.

## Results

### Sample

Women's ages ranged from 18–44 and almost all (94%) had completed secondary school or more (see Table 2). Women had been in a relationship with their current partner for an average of 4.5 years; however, only about one-quarter of them (26%) currently lived with their partner. Few women (5%) changed partners during the course of the study. On average, women were nearly six months pregnant at their first antenatal care visit. Almost 40% experienced some type of violence in their current relationship at some point prior to pregnancy. Women experienced an average of 1 act of physical IPV each month during pregnancy and nearly 2 acts of physical IPV each month postpartum (see Table 3). Women experienced an average of 2.25 acts of psychological IPV each month during pregnancy and reported a similar number of acts of psychological IPV in the postpartum period (see Table 3). The IPV variables and relationship power variable were significantly correlated with one another at p<.05 (see Table S1 and Table S2 in File S1 for these and other key correlations).

**Table 2:** Women's sociodemographic and reproductive characteristics at baseline (n = 1,447).

		*(mean, sd)*
Participant's age (years)		25.49 (5.35)
Length of relationship (years)		4.48 (4.09)
Gestational age (weeks)		24.21 (6.03)
		(n,%)
Live together		376 (25.98)
Education*	*Primary*	95 (6.57)
	*Secondary*	611 (42.25)
	*More than secondary*	740 (51.18)
HIV+ status at baseline		560 (38.70)
No. of prior pregnancies	*0*	507 (35.04)
	*1*	546 (37.73)
	*2 or more*	394 (27.23)
Pregnancy unintended**		1154 (80.00)
Experienced IPV in current relationship before pregnancy		545 (37.66)

*n = 1,446 due to missing data; **n = 1,440 due to missing data.

**Table 3:** Mean number of physical and psychological IPV events reported per month during pregnancy and the postpartum period^a^.

	During Pregnancy	Delivery to 14 weeks postpartum	14 weeks to nine months postpartum
Physical IPV	1.07 (4.51)	1.92 (7.42)	1.93 (7.78)
Psychological IPV	2.25 (6.22)	2.63 (7.63)	2.05 (6.87)

*^a^Data are expressed as (mean, sd).*

### Trajectories of physical IPV

Parameter estimates for the unconditional model (model with no covariates) describing the trajectory of physical IPV across pregnancy and the postpartum period are presented in Column 1, Table 4. The best model fit for physical IPV was a linear model specifying a random intercept and random slope with heteroscedastic residuals. The fixed effect for time was not significant (*B* = .009, p = .52), which means that, on average, trajectories of physical IPV tended to stay flat across the study period. However, significant random effects indicate that there was significant individual variability around this average trajectory. In particular, there was significant individual variability in initial levels of physical IPV assessed during pregnancy (i.e., trajectory intercepts; Var(B)  = .040, p<.0001) as well as in rates of change in physical IPV across the postpartum period (i.e., trajectory slopes; Var(B)  = .022, p<.0001). Further, there was a significant negative covariance between the random intercepts and slopes, which suggests that women who reported higher initial levels of physical IPV tended to report lower slope scores.

**Table 4:** Parameter estimates and standard errors for the unconditional and conditional models of physical IPV during pregnancy and the first nine months postpartum*,**.

	Unconditional	Conditional
	B (SE)	B(SE)
*Fixed effects*		
intercept	0.774 (.013)^a^	0.727 (.014)^a^
time	0.009 (.009)	0.003 (.014)
pre-pregnancy IPV	.	0.059 (.013)^a^
pre-pregnancy IPV*time	.	0.043 (.011)^a^
pre-pregnancy IPV*relationship power	.	−0.008 (.002)^a^
relationship power	.	−0.004 (.001)^a^
partner social support	.	−0.002 (.003)
relationship stress	.	0.007 (.005)
*Random effects*		
intercept variance	0.040 (.005)^a^	0.032 (.004)^a^
time variance	0.022 (.003)^a^	020 (.002)^a^
covariance (intercept with time)	−0.008 (.002)^b^	−0.008 (.002)^b^

a p<.0001, ^b^p<.05.

*residual errors were allowed to vary over time and were significant at each time point.

**conditional model controls for variation in weeks at each time point, age, cohabitation and treatment arm.

#### Testing of interactions

The set of three way interactions did not contribute significantly to the model (see Table 1, Set 1) indicating that the effect of pre-pregnancy IPV on trajectory slopes did not vary as a function of relationship power, partner social support or relationship stress. The set of two-way interactions with time (see Table 1, Set 2) was significant; the only significant individual interaction within this series was Pre-pregnancy IPV X Time. Finally, the set of two-way interactions with intercepts (see Table 1, Set 3) was also significant; the only significant individual interaction within this series was Pre-pregnancy IPV X Relationship Power.

Results from the final reduced conditional model for physical IPV (which retained the two significant interactions identified in the testing described above) are presented in Table 4, Column 2. Parameter estimates from this model suggest that pre-pregnancy IPV was associated with physical IPV trajectory intercepts (*B* = .059, p<.0001) and slopes (*B* = .043, p<.0001.); however, consistent with study hypotheses, the effect of pre-pregnancy IPV on trajectory intercepts was moderated by relationship power (*B* = −.008, p<.0001). Contrary to study hypotheses, neither partner social support nor relationship stress was associated with physical IPV trajectory intercepts or slopes.

**Figure 1:**
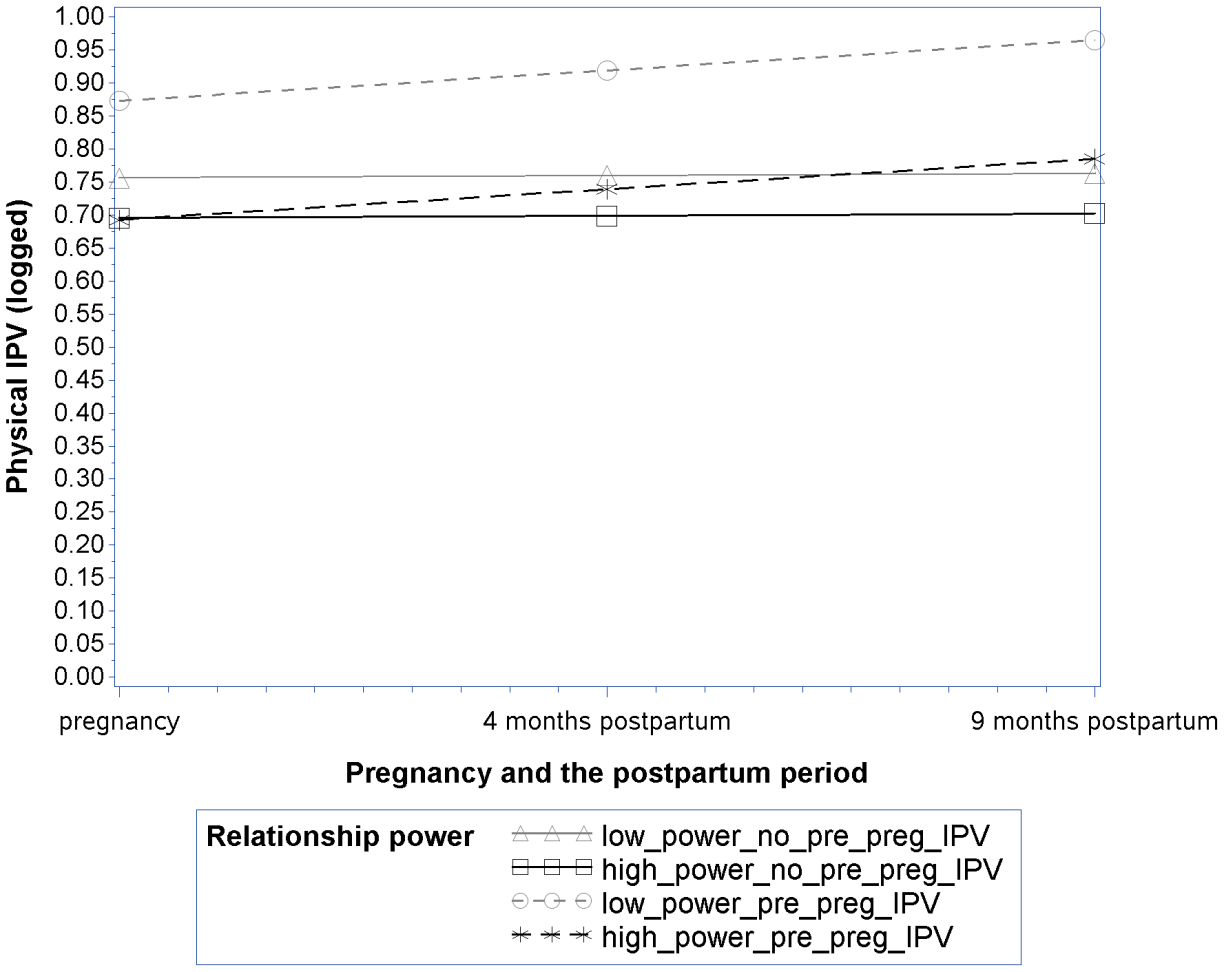
Physical IPV over time by relationship power and pre-pregnancy IPV. The displayed lines represent the predicted values estimated by the final model where relevant fixed effects are set at selected illustrative levels. “High” and “low” relationship power were defined as being one standard deviation above and below the sample mean on this variable respectively.

In order to visually illustrate key effects of our final reduced longitudinal model for physical IPV, we estimated and plotted mean trajectories predicted by the model at selected levels of relevant fixed effects for four groups of women: women with pre-pregnancy IPV and high power, women with no pre-pregnancy IPV and high power, women with pre-pregnancy IPV and low power, women with no pre-pregnancy IPV and low power (Figure 1). “High” and “low” relationship power were defined as being one standard deviation above and below the sample mean on this variable respectively. As shown in Figure 1, consistent with study hypotheses, the effect of pre-pregnancy IPV on initial levels of physical IPV (i.e., trajectory intercepts) was moderated by relationship power such that the association was weaker for women with high power than for women with low power (Pre-Pregnancy IPV X Relationship Power interaction). This can be seen by looking at the magnitude of the difference between the trajectory intercepts for low power women (gray lines) with and without pre-pregnancy IPV (large significant difference; *B* = .12, p<.0001) compared to the difference between the trajectory intercepts for high power women (black lines) with and without pre-pregnancy IPV (smaller insignificant difference; *B* = −.003, p = .88). Further, these differences extend through the postpartum period. In addition, regardless of relationship power, pre-pregnancy IPV also was significantly associated with sharper increases in physical IPV over time (trajectory slopes) as is reflected by the dashed lines in the figure (Pre-Pregnancy IPV X Time interaction).

### Trajectories of psychological IPV

Parameter estimates for the unconditional model (model with no covariates) describing the trajectory of psychological IPV across pregnancy and the postpartum period are presented in Column 1, Table 5. Similar to the unconditional model for physical IPV, the best model fit for psychological IPV was a linear model specifying a random intercept and random slope with heteroscedastic residuals. The fixed effect for time was not significant (*B* = −.027, p = .13), which means that, on average, trajectories of psychological IPV tended to stay flat across the study period. However, significant random effects indicate that there was significant individual variability around this average trajectory. In particular there was significant individual variability in initial levels of psychological IPV assessed during pregnancy (i.e., trajectory intercepts; (Var(B) = .053, p<.0001) as well as in rates of change in psychological IPV across the postpartum period (i.e., trajectory slopes; (Var(B) = .023, p<.0001).Further, there was a significant negative covariance between the random intercepts and slopes, which suggests that women who reported higher initial levels of psychological IPV tended to report lower slope scores.

**Table 5:** Parameter estimates and standard errors for the unconditional and conditional models of psychological IPV during pregnancy and the first nine months postpartum*,**.

	Unconditional	Conditional
	B (SE)	B (SE)
*Fixed effects*		
intercept (	0.858 (.017)^a^	0.847 (.038)^a^
time	−0.027 (.018)	−0.017 (.017)
pre-pregnancy IPV	.	0.144 (.014)^a^
pre-pregnancy IPV*relationship power	.	−0.010 (.002)^a^
relationship power	.	−0.006 (.001)^a^
relationship power*time	.	0.002 (.001)^c^
partner social support	.	−0.008 (.004)^b^
relationship stress	.	0.026 (.007)^b^
relationship stress*time	.	−0.012 (.005)^c^
*Random effects*		
intercept variance	0.053 (.008)^a^	0.042 (.007)^a^
time variance	0.023 (.004)^a^	0.026 (.004)^a^
covariance (intercept with time)	−0.012 (.004)^c^	−0.014 (.004)^c^

a p<.0001, ^b^p<.001, ^c^p<.05.

*residual errors were allowed to vary over time and were significant at each time point.

**conditional model controls for variation in weeks at each time point, age, cohabitation and treatment arm.

#### Testing of interactions

Similar to our findings for physical IPV, the set of three way interactions did not contribute significantly to the model (see Table 1, Set 1) indicating that the effect of pre-pregnancy IPV on trajectory slopes did not vary as a function of relationship power, partner social support or relationship stress. The set of two-way interactions with time (see Table 1, Set 2) was significant; the significant interactions within this series were Relationship Power X Time and Relationship Stress X Time. Finally, the set of two-way interactions with intercepts (see Table 1, Set 3) was also significant; the only significant individual interaction within this series was Pre-pregnancy IPV X Relationship Power.

Results from the final reduced conditional model for psychological IPV (which retained the three significant interactions identified in the testing described above) differ slightly from physical IPV and are presented in Table 5, Column 2. Parameter estimates from this model suggest that pre-pregnancy IPV was associated with psychological IPV trajectory intercepts (*B* = .144, p<.0001) and that relationship power was associated with psychological IPV trajectory slopes (*B* = .002, p<.05.) Further, consistent with study hypotheses, the effect of pre-pregnancy IPV on trajectory intercepts was moderated by relationship power (*B* = −.010, p<.0001). Contrary to study hypotheses, neither partner social support nor relationship stress moderated the association between pre-pregnancy IPV and psychological IPV during pregnancy and the first nine months postpartum. However, partner social support was associated with trajectory intercepts (*B* = −.008, p<.001), such that women who had higher levels of partner social support had lower levels of psychological IPV at pregnancy than women who had lower levels of partner social support. Similarly, relationship stress was associated with trajectory intercepts (*B* = .026, p<.001) and trajectory slopes (*B* = −.012, p<.05), such that women who had higher relationship stress had higher levels of psychological IPV at pregnancy, though levels declined over time.

**Figure 2:**
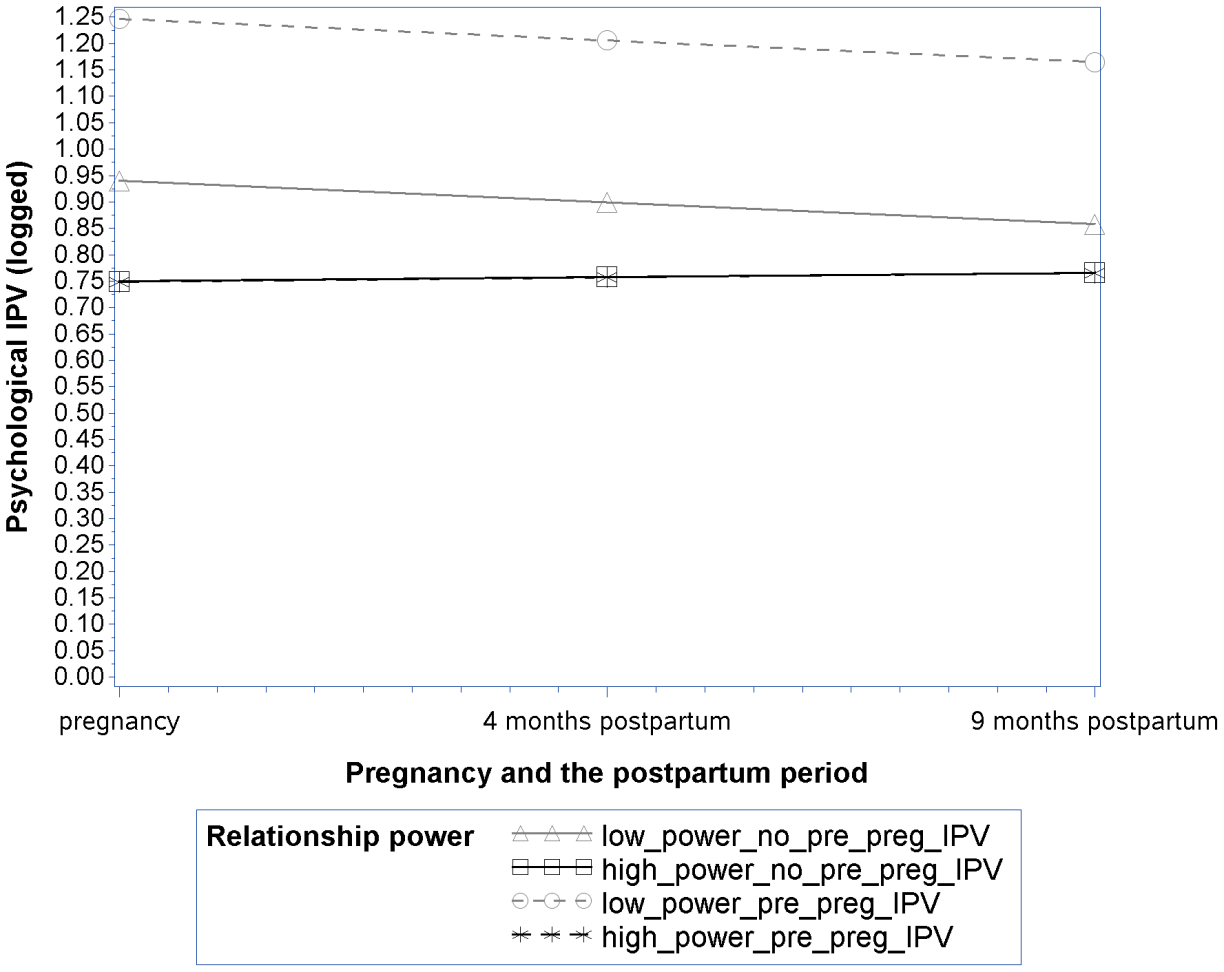
Psychological IPV over time by relationship power and pre-pregnancy IPV. The displayed lines represent the predicted values estimated by the final model where relevant fixed effects are set at selected illustrative levels. “High” and “low” relationship power were defined as being two standard deviations above and below the sample mean on this variable respectively.

As was done in the physical violence analyses, in order to visually illustrate key effects of our final reduced longitudinal model for psychological IPV, we estimated and plotted mean trajectories predicted by the model at selected levels of relevant fixed effects for four groups of women: women with pre-pregnancy IPV and high power, women with no pre-pregnancy IPV and high power, women with pre-pregnancy IPV and low power, women with no pre-pregnancy IPV and low power (Figure 2). “High” and “low” relationship power were defined as being two standard deviations above and below the sample mean on this variable respectively. As shown in Figure 2, consistent with study hypotheses, the effect of pre-pregnancy IPV on initial levels of psychological IPV (i.e., trajectory intercepts) was moderated by relationship power such that the association was weaker for women with high power than for women with low power (Pre-Pregnancy IPV X Relationship Power interaction). This can be seen by looking at the magnitude of the difference between the trajectory intercepts for low power women (gray lines) with and without pre-pregnancy IPV (large significant difference; *B* = .28, p<.0001) compared to the difference between the trajectory intercepts for high power women (black lines) with and without pre-pregnancy IPV (smaller insignificant difference; *B* = −.001, p = .97). Further, these differences extend through the postpartum period. In addition, while lower levels of power at baseline were associated with higher levels of psychological IPV (and this effect was even stronger for women who had experienced pre-pregnancy IPV), the fact that lower levels of power were associated with greater decreases in IPV over time (as reflected in the Relationship Power*Time interaction) may reflect a ceiling effect for women who started with higher levels of psychological IPV. This speculation is supported by the fact that the random intercept and random slope variances were negatively correlated.

## Discussion

The current study was methodologically innovative in its use of random coefficients multilevel growth curve modeling (1) to describe the mean trajectory of IPV over pregnancy and the first nine months postpartum and (2) to examine the association between pre-pregnancy IPV and trajectories of IPV and to determine whether *resources* and *experiences* within the relationship moderated the association between pre-pregnancy IPV and IPV during pregnancy and the first nine months postpartum. The mean trajectory for both types of IPV was flat which suggests that, on average, there was not significant change in levels of IPV over pregnancy and the first nine months postpartum. However, there was significant variability in levels of both types of IPV at pregnancy and in rates of change in IPV over pregnancy and the postpartum period. One of the proposed *resources* – relationship power – moderated the effects of pre-pregnancy IPV on trajectory intercepts, which affected women's levels of physical and psychological IPV at pregnancy and subsequently, their trajectories of IPV over time. None of the proposed *resources* and *experiences* moderated the effects of pre-pregnancy IPV on trajectory slopes, or rates of change in IPV over time. However, there were independent main effects for partner social support and relationship stress. Specifically, partner social support was negatively associated with levels of psychological IPV at pregnancy, and relationship stress was positively associated with levels of psychological IPV at pregnancy and with rates of change in psychological IPV over time.

To date, there has been no longitudinal study of whether and how IPV changes during pregnancy and the postpartum period for South African women. Our finding that on average IPV did not change over pregnancy and the postpartum period is consistent with one other longitudinal study on IPV among high risk women in the United States during this time period [9] and inconsistent with a different U.S. based longitudinal study of IPV during this time period [11]. These inconsistent findings may be because of differences in study design or because IPV changes during pregnancy and the postpartum period for some populations and not others. Additional longitudinal research will allow us to discern the patterns of IPV before, during and after pregnancy in other settings in sub-Saharan Africa and globally [35], [36].

Although the average trajectory of IPV did not change during pregnancy and the postpartum period, our finding that there was substantial variability across women in their experiences of IPV during this time period is consistent with existing cross sectional literature in other settings [8], [10], [12], [37]. The variability observed across women suggests some women do experience increases in IPV during pregnancy and the postpartum period. Interventions to identify and target these women are needed.

Further, although our hypotheses that specific relationship characteristics would modify the association between pre-pregnancy IPV and IPV during pregnancy and the postpartum period were not fully supported, our findings make a substantial contribution to the literature by increasing our understanding of how *resources* and *experiences* within the relationship can protect women from IPV, particularly during pregnancy. Consistent with study hypotheses, higher relationship power protected women with pre-pregnancy IPV from both physical and psychological IPV during pregnancy. Further, higher relationship power continued to confer some protection in the postpartum period, although physical IPV began to increase for women with pre-pregnancy IPV again during this time. And finally, the detrimental effects of pre-pregnancy IPV were exacerbated for women with lower relationship power such that the effects of pre-pregnancy IPV were stronger for women with low power than those with high power at each time point in the study. These findings are consistent with a “dual-risk” model which posits that negative effects of different factors can be synergistic in terms of one's susceptibility to IPV [38], [39]. There was no support for the hypothesis that partner social support would mitigate the association between pre-pregnancy IPV and IPV during pregnancy and the postpartum period. This lack of support is in contrast to the findings of Sampson and Laub's study that men who entered into marriage characterized by stronger relationships at marriage are less likely to continue to engage in criminal activity than men who entered into marriage characterized by weaker relationships [16]. It may be that relationship qualities have a more profound impact on trajectories at the transition of marriage than the transition to parenthood. Another possibility is that partner social support may function differently across relationships such that for some individuals support may be a positive characteristic, whereas for others it may be negative. For example, support may be financial or material in nature and some partners may provide this support as a means of control. Nonetheless, there was an independent effect of partner social support on levels of psychological IPV at pregnancy, such that women with higher levels of partner social support had lower levels of psychological IPV at pregnancy than women with lower levels of partner social support. The same effect was not observed with physical IPV, which suggests that the etiology of psychological IPV during this time may be distinct from physical IPV and warrants further study.

There was also no support for the hypothesis that relationship stress strengthened the association between pre-pregnancy IPV and IPV during pregnancy and the postpartum period. The lack of association may be because our measurement of relationship stress captured only one dimension of stress: the presence or absence of specific life events. Other research suggests stress is a multidimensional construct and that one's perception of life events (as stressful or not) are also related to IPV risk [30]. It is possible that it is not the stress itself that matters, but rather one's perceptions of life events as stressful. These perceptions might moderate the association between pre-pregnancy IPV and IPV during pregnancy and the postpartum period. On the other hand, there was an independent positive effect between relationship stress and psychological IPV during this time. This effect may have been observed because psychological IPV, which was much more prevalent in our sample than physical IPV, is more sensitive to relationship stress [40]. Alternatively, it may that the association between relationship stress and physical IPV depends on one's beliefs about the acceptability of perpetrating physical IPV during this time period or on the norms regarding the acceptability of perpetrating IPV [32]. Our findings are consistent with a study among women in the United States which found that particular pregnancy-related factors (parity and mistimed pregnancies) that were hypothesized to be associated with stress were associated with cessation of IPV during the study period [20]. However, our study and Jasinksi's study both assumed that these life events were related to stress rather than directly measuring stress itself. Additional research on how to best conceptualize and measure stress in the South African context and on beliefs and attitudes about IPV during pregnancy and the postpartum time period is needed.

The current study has three limitations. First, all three moderators in our study were measured as time-stable. It is possible that there was a lack of an interaction over time because the effects of *resources* and *experiences* on the association between pre-pregnancy IPV and trajectories of IPV are more proximal. Future studies should model *resources* and *experiences* as time-varying, which would allow us to assess whether increases or decreases in an individual's *resources* and *experiences* at each time point is concurrently associated with stronger or weaker associations in the relationship between pre-pregnancy IPV and IPV at that same time point. A second limitation is that our sample was comprised only of women, despite the fact that our moderator variables were really assessing *resources* and *experiences* of the relationship. Future research is needed to understand how men describe their levels of relationship power, social support and relationship stress and during this time. A third limitation is that the measurement of pre-pregnancy violence within the relationship was a lifetime measure of IPV within the current relationship prior to pregnancy. Such measurement may have been particularly susceptible to recall bias. Future research should ask women specifically about their experiences of IPV within their current partnership in the year preceding pregnancy. Despite these limitations, this is the first study to examine trajectories of IPV among South African women during pregnancy and the postpartum period and the findings have significant implications for IPV interventions during pregnancy and the postpartum period. The finding that the mean trajectory was flat suggests that, on average, women's risk for IPV does not increase or decrease from pregnancy into the postpartum period. However, the significant variability across women in their individual trajectories suggests that some women do face increased or decreased risk of IPV during this time period. Further, we identified particular modifiable relationship characteristics associated with risk of IPV during this time. Interventions that include components designed to maximize *resources* and minimize negative *experiences* during pregnancy may be especially effective at reducing psychological violence during pregnancy since relationship power, partner social support and relationship stress were each associated with psychological IPV in pregnancy. Interventions that target women's relationship power may also reduce women's risk of physical IPV during both pregnancy and the postpartum period.

Furthermore, research and interventions outside of the healthcare sector are also needed to address IPV during pregnancy and the postpartum period. While there is increased attention to the importance of engaging men to reduce violence and HIV risk [41], there has been limited attention to men and their role as partners and fathers during pregnancy and the postpartum period. Their role in the reduction of IPV during this time should not be overlooked.

In conclusion, women with a history of pre-pregnancy IPV and lower relationship power were at heightened risk of both physical and psychological IPV during pregnancy and the postpartum time period; further, women with lower partner social support and higher relationship stress were at heightened risk of psychological IPV during this time, irrespective of past experiences of IPV within their relationship. Women's active engagement with the health care sector during and following their pregnancy represents a prime opportunity for screening and intervention. Given the negative health ramifications of IPV during this time period for both women and their children, interventions that reduce women's risk of IPV are urgently needed.

## Supporting Information

File S1 Table S1, Correlation matrix between physical IPV and relationship factors. Table S1 presents the correlations between physical IPV and the three relationship factors of interest. Table S2, Correlation matrix between psychological IPV and relationship factors. Table S2 presents the correlations between psychological IPV and the three relationship factors of interest.
(DOCX)

Data Set S1 This file presents the data necessary to replicate the analysis in the manuscript.
(ZIP)
