# Supplementary material for: Relationship Factors and Trajectories of Intimate Partner Violence among South African Women during Pregnancy and the Postpartum Period
Source: PLoS One. 2014 Sep 30;9(9):e106829. doi: 10.1371/journal.pone.0106829 (PMC4182034; doi:10.1371/journal.pone.0106829)
Supplement: File S1 — Table S1, Correlation matrix between physical IPV and relationship factors. Table S1 presents the correlations between physical IPV and the three relationship factors of interest. Table S2, Correlation matrix between psychological IPV and relationship factors. Table S2 presents the correlations between psychological IPV and the three relationship factors of interest. (DOCX) [file pone.0106829.s001.docx]

| **Table S1. Correlation matrix between physical IPV and relationship factors** | | | | | | | |
| --- | --- | --- | --- | --- | --- | --- | --- |
|  | IPV pregnancy | IPV 4 months postpartum | IPV 9 months postpartum | Pre-pregnancy IPV | Relationship power | Partner social support | Relationship stress |
| IPV pregnancy | 1.00 | .38*** | .28*** | .15*** | -.25*** | -0.03 | .05^ |
| IPV 4 months post |  | 1.00 | .49*** | .18*** | -.17*** | 0.00 | 0.03 |
| IPV 9 months post |  |  | 1.00 | .21*** | -.19*** | 0.00 | 0.04 |
| Pre-pregnancy IPV |  |  |  | 1.00 | -.18*** | 0.03 | 0.01 |
| Relationship power |  |  |  |  | 1.00 | .04^ | -0.09 |
| Partner social support |  |  |  |  |  | 1.00 | -.20*** |
| Relationship stress |  |  |  |  |  |  | 1.00 |
| ***p<.0001, ^p<.10 | | |  |  |  |  |  |

| **Table S2. Correlation matrix between psychological IPV and relationship factors** | | | | | | | |
| --- | --- | --- | --- | --- | --- | --- | --- |
|  | IPV pregnancy | IPV 4 months postpartum | IPV 9 months postpartum | Pre-pregnancy IPV | Relationship power | Partner social support | Relationship stress |
| IPV pregnancy | 1.00 | 0.27*** | 0.23*** | 0.23*** | -0.28*** | -0.08* | 0.11*** |
| IPV 4 months post |  | 1.00 | 0.47*** | 0.15*** | -0.15*** | -0.01 | 0.09* |
| IPV 9 months post |  |  | 1.00 | 0.24*** | -0.18** | -0.02 | 0.03 |
| Pre-pregnancy IPV |  |  |  | 1.00 | -0.18*** | 0.03 | 0.01 |
| Relationship power |  |  |  |  | 1.00 | 0.04^ | -0.09** |
| Partner social support |  |  |  |  |  | 1.00 | -0.20*** |
| Relationship stress |  |  |  |  |  |  | 1.00 |
| ***p<.0001, **p<.001, *p<.05, ^p<.10 | | |  |  |  |  |  |
